# A multi-site study of implementing a school-based health promotion intervention in Sweden – changes in readiness and implementation outcomes

**DOI:** 10.1186/s12889-026-29302-z

**Published:** 2026-09-08

**Authors:** Kristi Sidney Annerstedt, Lydia Kwak, Susanne Andermo, Emma Patterson, Åsa Norman, Sara Raposo, Liselotte Schäfer Elinder

**Affiliations:** 1 Department of Global Public Health, Karolinska Institutet, Solna, Sweden; 2 Institute for Environmental Medicine, Karolinska Institutet, Solna, Sweden; 3 Department of Neurobiology, Care Sciences and Society, Karolinska Institutet, Huddinge, Sweden; 4 The Swedish School of Sport and Health Sciences (GIH), Stockholm, Sweden; 5 Swedish Food Agency, Uppsala, Sweden; 6 Department of Women’s and Children’s Health, Uppsala University, Uppsala, Sweden; 7 Department of Clinical Neuroscience, Karolinska Institutet, Solna, Sweden; 8 Centre for Epidemiology and Community Medicine (CES), Region Stockholm, Sweden

**Keywords:** Readiness, Acceptability, Appropriateness, Feasibility, Hybrid type 3 study, Implementation, Schools

## Abstract

**Background:**

Schools are a key setting for child health promotion, yet implementing school-based programs, particularly those involving parents, remains challenging. Organizational readiness and early implementation outcomes, acceptability (ACC), appropriateness (APP), and feasibility (FEAS), capture how stakeholders perceive the implementability of new interventions and engage with them. These perceptions may evolve during implementation, but evidence on how they change over time and relate to one another in real-world school settings is limited. This study examined these changes among school staff during the first year of implementing the Healthy School Start program in schools in three Swedish municipalities (M).

**Methods:**

Validated questionnaires were used to assess readiness, ACC, APP, and FEAS pre- and post-intervention to examine how these implementation variables varied with direct experience of the intervention. Data were collected from 39 school principals, 72 teachers, and 40 school nurses between September 2021 and June 2023. Multilevel linear regression models with random intercepts for school and individual examined pre-post changes and interactions by municipality and professional group.

**Results:**

Readiness, ACC, and APP declined significantly from pre- to post-intervention in the total sample (readiness: β = −2.54, *p* = 0.032; ACC: β = −1.31, *p* = 0.004; APP: β = −1.12, *p* = 0.022), while FEAS remained stable (β = −0.30, *p* = 0.501). M2 schools reported lower baseline scores than M1 schools on ACC, APP, and FEAS. M1 schools showed significant declines across all four outcomes, while M3 schools remained stable. FEAS was the only outcome for which pre-post change differed significantly across municipalities (time × municipality interaction: χ²(2) = 9.56, *p* = 0.008), driven by a decline in M1. No significant differences were observed between professional groups, although school nurses reported the lowest readiness and feasibility scores. Baseline readiness was strongly correlated with ACC (ρ = 0.66), APP (ρ = 0.67), and FEAS (ρ = 0.73; all *p* < 0.001).

**Conclusion:**

Perceptions of implementability generally declined with feasibility remaining stable overall except for one municipality showing a significant decline. These findings run counter to assumptions that exposure alone improves implementation outcomes, and suggest that perceptions are shaped by organizational context, underscoring the importance of addressing contextual barriers both before and during implementation.

**Trial registration:**

Registered prospectively at ClinicalTrials.gov ID: NCT04984421, registered July 30, 2021.

**Supplementary Information:**

The online version contains supplementary material available at https://doi.org/10.1186/s12889-026-29302-z.

## Background

Schools are a key setting for delivering health promotion interventions for children aged 6–12 years [1, 2]. However, implementing school-based programs, particularly those involving parents, remains challenging in routine practice [3, 4] since some school principals are uncertain about whether it is the role of the school to provide health guidance to parents in addition to teaching children [5]. There also seems to be a ‘responsibility conflict’ between the school and parents [6]. Other barriers are competing curriculum demands, lack of planning time, limited resources, and insufficient leadership support [7]. Within this context, school nurses play a central role in health promotion delivery yet often report feeling overburdened and isolated in this responsibility [5, 8]. These challenges highlight the importance of understanding how different school staff perceive and respond to the implementation of new interventions [9, 10].

Organizational readiness for change refers to a shared psychological state reflecting commitment to change and collective efficacy among members of an organization, capturing how prepared individuals feel to engage with a new intervention [9]. When readiness is high, members are more likely to initiate change, and display more cooperative behavior, which overall results in more effective implementation of the proposed change. Early implementation outcomes (e.g., acceptability (ACC), appropriateness (APP), and feasibility (FEAS)) reflect how stakeholders perceive an intervention in practice, including whether it is agreeable, suitable, and workable within a given setting, as described in the Proctor Implementation Outcomes Framework [11].

An organizationally receptive setting increases the likelihood of successful integration and adoption of a new program, supporting its perceived ACC and APP within the organizational setting. However, while a program may be found acceptable by stakeholders and appropriate for a given setting, it may not be feasible, typically due to a lack of resources or competence of the providers. While conceptually distinct, evidence suggests that these implementation outcomes are highly correlated and could serve as proxies for each other [12]. Understanding readiness, ACC, APP and FEAS early in the research process could help ensure that interventions and implementation strategies are optimized, fit with ‘end-users’ preferences, and can be scaled up and sustained.

Importantly, both readiness and implementation outcomes are not static but may evolve over the course of implementation and exposure to implementation processes, training, and real-world delivery can shape perceptions of preparedness, benefit, and feasibility [13]. Assessing these constructs before and after implementation therefore allows examination of how experience with an intervention influences implementation-related perceptions, and how these may differ across professional groups. Despite increasing interest in implementation processes, there is limited empirical research examining how readiness and early implementation outcomes change over time in real-world school settings, particularly in the context of health promotion interventions [14]. In addition, little is known about how these perceptions are related to one another as implementation progresses [15].

The present study was conducted during the first year of scaling up the universal Healthy School Start (HSS) program in compulsory pre-school class or grade one across schools in three different municipalities with higher health needs in the Stockholm Region in Sweden [16]. The HSS program is an evidence-based family support intervention targeting diet and physical activity, with demonstrated effectiveness in improving health behaviors and reducing obesity risk [17–20]. These results are in line with other studies showing that school-based health promotion programs with direct parental involvement, especially for children up to 12 years which simultaneously address multiple behaviors in the school and home environments, can be effective [3, 21, 22]. In Sweden, the large majority of compulsory schools (over 80%) operate under local municipal governance and vary widely in staff qualifications and resource allocation, depending on socio-demographic profiles. These contextual differences are known to influence implementation processes within school settings [10, 23, 24]. All compulsory schools in Sweden, whether municipal or independent, follow the national curriculum and the Education Act, which mandate health promotion as a school responsibility. The HSS program was designed to align with this legislation and the National Guidelines for School Health Services to facilitate implementation.

To address the gap identified above, this study assessed readiness and early implementation outcomes (ACC, APP, FEAS) at the individual level before and after the delivery of a one-year family support intervention during the initial phase of a municipal scale-up study. By examining changes over time and associations between these variables, this study aims to better understand how implementation-related perceptions develop during program delivery. The specific aims of this study were to: (1) investigate changes in perceived readiness, ACC, APP and FEAS from pre- to post-intervention in schools in three municipalities and in different professional groups (principals, teachers, and school nurses); and (2) examine the correlations between baseline readiness and ACC, APP, and FEAS.

## Methods

### Intervention

The HSS program is delivered in schools by a team led by the principal or vice principal in collaboration with the school nurse and teachers. It includes four components: (1) A brochure with health information sent home to the parents, (2) health talks using motivational interviewing given by the school nurse with the parents, (3) nine classroom lectures led by teachers with homework assignments to be completed with the child’s parents or guardians (henceforth referred to as parents), and (4) an online risk test with eight questions for type 2 diabetes done by parents. The program was implemented starting in 2021 in a hybrid type 3 implementation-effectiveness study called IMPROVE: IMplementation and evaluation of the school-based family support PRogram a Healthy School Start to promote child health and prevent OVErweight and obesity [16]. The IMPROVE study evaluated two bundles of implementation strategies (Basic and Enhanced) on the primary outcome of fidelity to the four program components after two years of implementation. The main outcome paper has recently been published [25].

### Study context

In Sweden, the National Guidelines for school health care [26] mandate at least three routine general health checks with every child during primary school. The first of these takes place in the compulsory preschool class (child aged 6–7 years) or first grade (child aged 7–8 years) and includes the parents. The HSS program was implemented in the same year as the routine health check with parents was scheduled: preschool class in Municipality 1 (M1) and Municipality 2 (M2) and in first grade in Municipality 3 (M3). Relevant characteristics of the three municipalities are shown in Table 1.

**Table 1:** Description of the population, schools and health in involved municipalities (M)

	Sweden	M1	M2	M3
Population characteristics				
Number of inhabitants^1^	10 400 000	113 951	50 273	101 209
Median income per year (SEK)^1^	460 000	333 318	306 861	288 880
Proportion with employment (%)^1^	68.8	79	77.3	75.3
Proportion unemployed (%)^1^	7.4	7.4	9.1	11.4
Proportion with ≥ 3 years university education 25–64 years (%)^1^	30.3	32.4	21.9	22.3
Proportion of inhabitants with ≤ 9 years of schooling^1^ 25–64 years (%)	10	10	14	17
Proportion of foreign-born inhabitants (year 2021)^1^ (%)	20.0	30.8	36.2	42.4
School characteristics				
Proportion of teachers with certificate (%)^2^	71.0	74.5	59.9	66.7
Number of eligible public primary schools	Na	25	13	19
Number of schools adopting the program	Na	15	13	19
School rank out of 290 municipalities (combination of teachers’ salary, teachers’ health and teacher density)^3^	Na	214	219	232
Density of students per teacher (rank out of 290 municipalities)^3^	Na	288	251	200
Health Indicators				
Overweight (not obesity) 4-year olds born 2017 (%)^4^	10.9	9.4	11.7	11.4
Obesity 4-year olds born 2017 (%)^4^ or 2016^5^	2.9	4.1	5.3	5.1
3-year olds with caries born 2019 (%)^4^	Na	3.7	5.4	3.3
Overweight and obesity in individuals ≥ 16 years (self-reported) (%)^6,7^	51.0	51.3	53.5	56.1
Diabetes in individuals ≥ 16 years (self-reported) (%)^6,8^	4.4–5	3.3	6	6.1

^1^Central Bureau of Statistics – Statistiska Centralbyrån SCB. Kommuner i siffror [Internet]. Stockholm: SCB; 2021/2022; ^2^Skolkollen.se 2023; ^3^Swedish Teacher’s Union – Lärarförbundet. Bästa skolkommun - resultat och historik [Internet]. Stockholm: Lärarförbundet; 2021. The ranking is based on quality with determinants such as amount of resources, number of children in preschool class, educated and healthy teachers, teachers per student, and student gradings. Low number corresponds to high ranking. ^4^Barnhälsovårdens Årsrapport 2022 (Yearly report Child Health Care), Region Stockholm. ^5^Miregård et al. Acta Paediatrica 2023 ^6^Folkhälsokollen.se, Region Stockholm, ^7^Public Health Agency of Sweden, ^8^Diabetics Association in Sweden

### Pre-implementation phase

In 2020, the research team contacted eight municipalities with a high prevalence of childhood obesity among 4-year-old children in Region Stockholm to explore their interest in participating in the IMPROVE study. Three central administrative leaders from the municipalities expressed interest. After an introduction to the program, two municipalities agreed to participate while the third delayed their participation by one year due to the ongoing COVID-19 pandemic. No schools were closed in Sweden during the pandemic. Prior to the start of the program in 2021, key implementation determinants were identified in workshops together with school personnel, according to the domains of the Consolidated Framework for Implementation Research [27]. Through a consensus process with stakeholders, two bundles of implementation strategies were identified and tailored to address context-specific determinants as described in the study protocol [16]. A timeline of the stakeholder engagement activities after recruitment at the municipality and school level and training of school personnel can be found in Additional file 1.

School principals were informed about the municipality’s intention to implement the program in all public schools, which they then communicated to their personnel. If a school had special reasons or circumstances such as staff shortages, the principal had the possibility to decline participation. A written agreement was signed between the municipalities and Karolinska Institutet to conduct the IMPROVE study during 2021 to 2024. Introductory meetings were given online for about one hour separately for each professional group (principals, teachers and school nurses) and participants were given the opportunity to ask questions. A website was developed for the program in Swedish and included all necessary information and program materials. Lectures in public health for school personnel given by experts in the area were available on the website. The program material including the implementation manual and the teacher’s manual was made available to school personnel two to three months before the start of the program.

### Study participants

The total number of participating schools was 15/25 (60%) in M1, 13/13 (100%) in M2 and 19/19 (100%) in M3. All school personnel working with the HSS program were invited to participate in the IMPROVE study through online or in-person information meetings, followed by an invitation sent via email. We used data collected from school leadership (i.e., principals and vice principals), school nurses and teachers, all referred to as school personnel henceforth [16]. We used an open cohort design since some teachers and a few school nurses started or ended their employment during the study period.

### Data collection and instruments used

Acceptability (ACC) is the perception among implementation stakeholders that a given intervention or innovation is agreeable, palatable, or satisfactory. Appropriateness (APP) expresses the perceived fit, relevance, or compatibility of the intervention for a given setting, provider, or consumer; and/or perceived fit of the intervention to address a particular problem. Feasibility (FEAS) is defined as the extent to which a new intervention can be successfully carried out within a given setting [11]. ACC, APP, and FEAS were assessed with a short and validated tool [12] translated to Swedish (Additional file 2). Each outcome consists of four items answered on a 5-point Likert scale with a total score between 4 and 20 points per outcome. Readiness was assessed with a modified version of the Organizational Readiness for Implementing Change (ORIC) tool developed by Shea et al. [28] and adapted into a *Leader Readiness to Implement Tool* (LRIT) answered by school principals and a *Staff Readiness to Implement Tool* (SRIT), answered by school nurses and teachers (Additional file 3). The questions were translated to Swedish and back translated to check for accuracy and minor adjustments were made. The respondent answers for him/her-self instead of on behalf of the school (all items start with “I who work here…” instead of “People who work here.”) as in the original ORIC scale. LRIT and SRIT consist of 13 items each answered on a 5-point Likert scale as follows: 0 = Disagree, 1 = Somewhat disagree, 2 = Neutral, 3 = Somewhat agree, and 4 = Agree.

Data collection was performed by electronic questionnaires using the secure web platform Research Electronic Data Capture (REDCap) between September 2021 and June 2023. An informational email was sent to all invited school personnel detailing the study and consent to participate. A link to the survey was included at the end of the email. The survey had three parts: a section with sociodemographic questions, the ACC, APP, and FEAS questionnaires, and lastly the LRIT and SRIT questionnaires. School personnel answered the questionnaires individually online during working hours at their school. Responses were collected after the HSS program had been introduced to the school personnel, but before the implementation had started in September-October and again post-intervention in May-June the following year. Automatic reminders were scheduled and sent weekly through REDCap up to three times after the initial invitation.

Organizational changes in the school were documented. This included changes within the school leadership (principal or vice principal), school nurse, teachers or other school reorganizations. The data was collected during visits made to each school in April-May 2022 (in M1 and M2) and April-May 2023 (M3) to review the performance of implementation strategies and through consultations with the municipality administration. It was hypothesized that organizational changes could impact on the outcomes of interest. Organizational instability was defined as any change to school personnel implementing the HSS program. The school was coded as 1 if there was school personnel turnover and 0 if there was no school personnel turnover.

### Statistical analysis

As this was a secondary analysis of data from the IMPROVE trial, the sample size was determined by the parent trial and no a priori power calculation was performed for the present study. A summative score was created for readiness (leadership and staff combined) and ACC, APP, FEAS respectively at pre- and post-intervention time points. Normality of outcome distributions was assessed using the Shapiro–Wilk test. Given the test indicated non-normal distributions for all four outcomes, descriptive statistics were presented as medians and interquartile ranges (IQR).

Changes from pre- to post-intervention were examined using multilevel linear regression models with up to two observations per participant. The outcome was the score at each time point, and the primary predictor was a binary time variable (0 = pre, 1 = post). Municipality and professional group were included as fixed effects to adjust for contextual differences. Random intercepts for school and for individuals (nested within school) accounted for clustering and repeated measurements. To assess whether pre-to-post change differed across municipalities or professional groups, interaction terms between time and municipality, and between time and professional group, were included in the model. Both interaction terms were fitted simultaneously in a single model for each outcome, rather than in separate models, to provide accurate variance estimation and to test both interactions against the same underlying model. The joint significance of each interaction was tested using Wald tests, and subgroup-specific changes were estimated as marginal effects from the same model. For baseline comparisons between municipalities, we report comparisons of M2 to M1 for each outcome. M1 and M2 began implementation at the same time and under the same pandemic conditions, so baseline differences between them reflect pre-existing municipality characteristics rather than differences in the implementation context. Since M3 began one year later under more typical conditions, baseline comparisons involving M3 are confounded by the timing lag and are therefore not reported; municipality-specific baseline values for all three municipalities are provided in Supplementary Table S1. Given the non-normal distribution of outcome scores, robust standard errors clustered at the school level were used throughout to address non-normality and potential heteroscedasticity. Models were fitted in Stata using the mixed command with vce(robust). Model assumptions, including normality of residuals and homoscedasticity, were assessed through visual inspection of Q-Q plots and residuals-vs-fitted plots. A p-value < 0.05 was considered statistically significant. Across the four implementation outcomes, multilevel models included 157–161 individuals nested within 47 schools and 248–262 observations, depending on item-level missingness (mean ~ 5 observations per school; mean 1.6 observations per individual). The mean of 1.6 observations per individual reflects that a proportion of participants provided data at only one time point; 151 individuals completed the baseline survey, 111 the post-intervention survey, and 81 provided both. Since participants with a single observation contribute to fixed-effect estimation but not to within-person variance, the individual-level random intercept was primarily identified by participants with data at both time points. Spearman rank correlations were calculated between individual baseline Readiness scores and baseline ACC, APP, and FEAS scores, given the non-normal distribution of the data. Bootstrap 95% confidence intervals (1,000 replications, clustered at the school level) were estimated to account for the clustered data structure. Scatter plots display the relationships between baseline readiness and baseline ACC, APP, and FEAS.

## Results

**Fig. 1:**
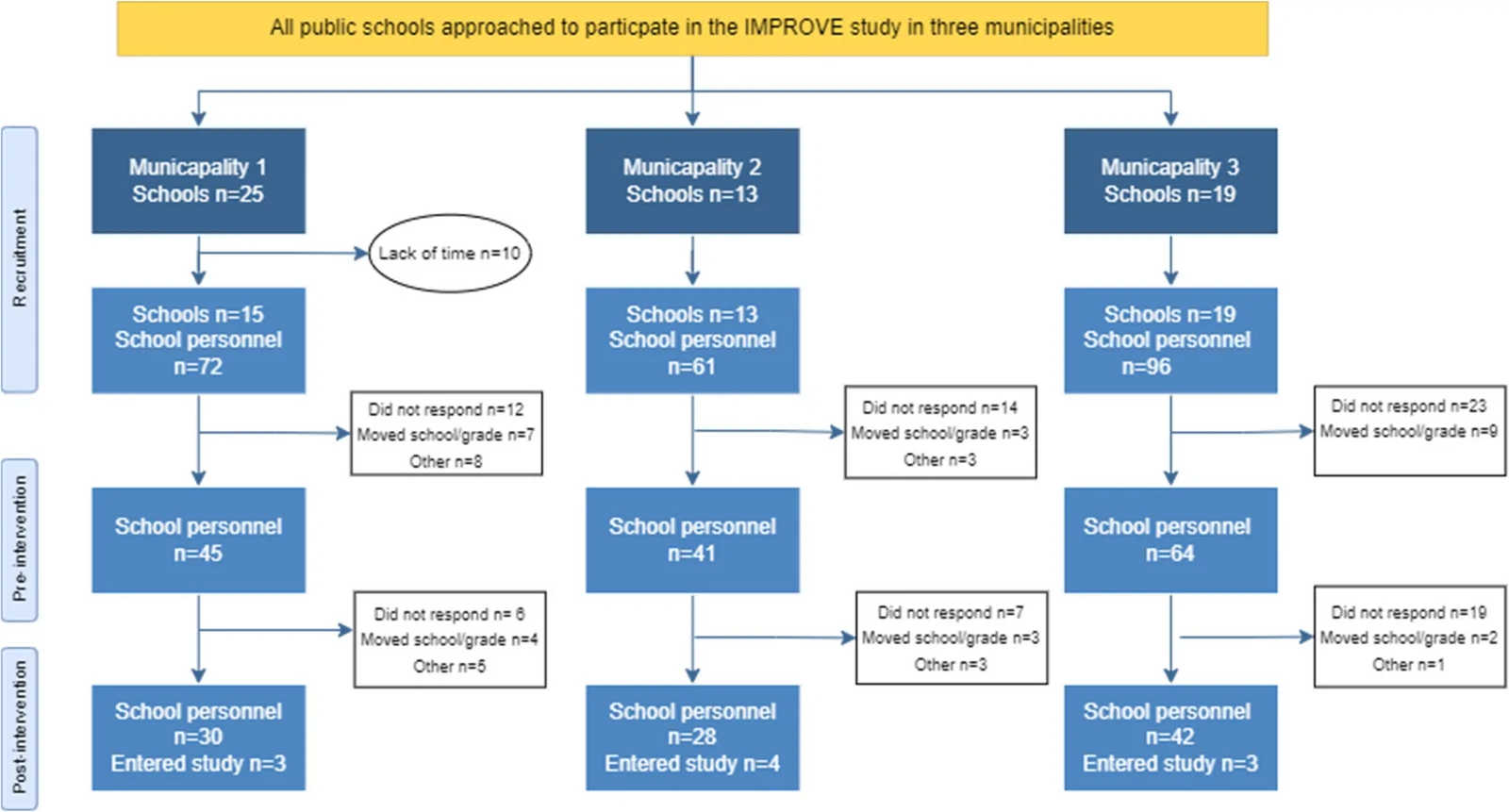
Flow chart describing the process of enrolling schools and personnel in the three municipalities

Figure 1 illustrates the flow of school and personnel enrollment across the study period. A total of 57 schools were eligible for inclusion; 10 schools in Municipality 1 (M1) declined participation, citing organizational changes or staff shortages. Of 229 eligible personnel across 47 schools, 151 (66%) completed baseline survey and of those 111 (74%) completed the post-intervention survey. The sample was predominantly female (94%) with a mean age of 46.4 years (Table 2). Professional roles comprised 48% teachers (*n* = 72), 26% school nurses (*n* = 40), and 26% principals (*n* = 39). Personnel in M1 reported significantly more years of work experience than those in M2 and M3 (*p* = 0.02). Other demographic characteristics did not differ significantly between municipalities. Organizational instability during the implementation period affected 40% of schools in M1 (6/15), 54% of schools in M2 (7/13), and 16% of schools in M3 (3/19). All schools in M3 continued the HSS program after year one, compared with partial continuation in M1 and discontinuation in M2.

**Table 2:** Characteristics of school personnel pre-intervention by municipality

Participant characteristics	Total*n* = 151	Municipality 1*n* = 45	Municipality 2*n* = 41	Municipality 3*n* = 65	*P*-value
Age mean years (SD)	46.4 (9.7)	48.5 (6.3)	46.4 (9.9)	45.2 (11.4)	0.22^*^
Female n (% in municipality)	142 (94)	41 (91)	40 (98)	61 (94)	0.45^**^
Professional title					
Principal/Vice-Principal	39 (26)	11 (24)	11 (27)	17 (26)	0.89^**^
Teachers n (%)	72 (48)	23 (51)	17 (41)	32 (49)	
School Nurses n (%)	40 (26)	11 (24)	13 (32)	16 (25)	
Work experience in the field median years (IQR)	7 (3,15)	11 (5,20)	5 (2,15)	6 (2,10)	0.02^†^
Work experience in the current school median years (IQR)	4 (2,7)	4 (2,6)	4 (2,8)	3 (1,6)	0.56^†^

*SD* Standard deviation, *IQR* Interquartile range

*ANOVA; **Pearson’s Chi-Squared test; ^†^Kruskal-Wallis tests used to test differences between municipalities

### Changes in readiness, acceptability, appropriateness, and feasibility from pre- to post-intervention

**Fig. 2:**
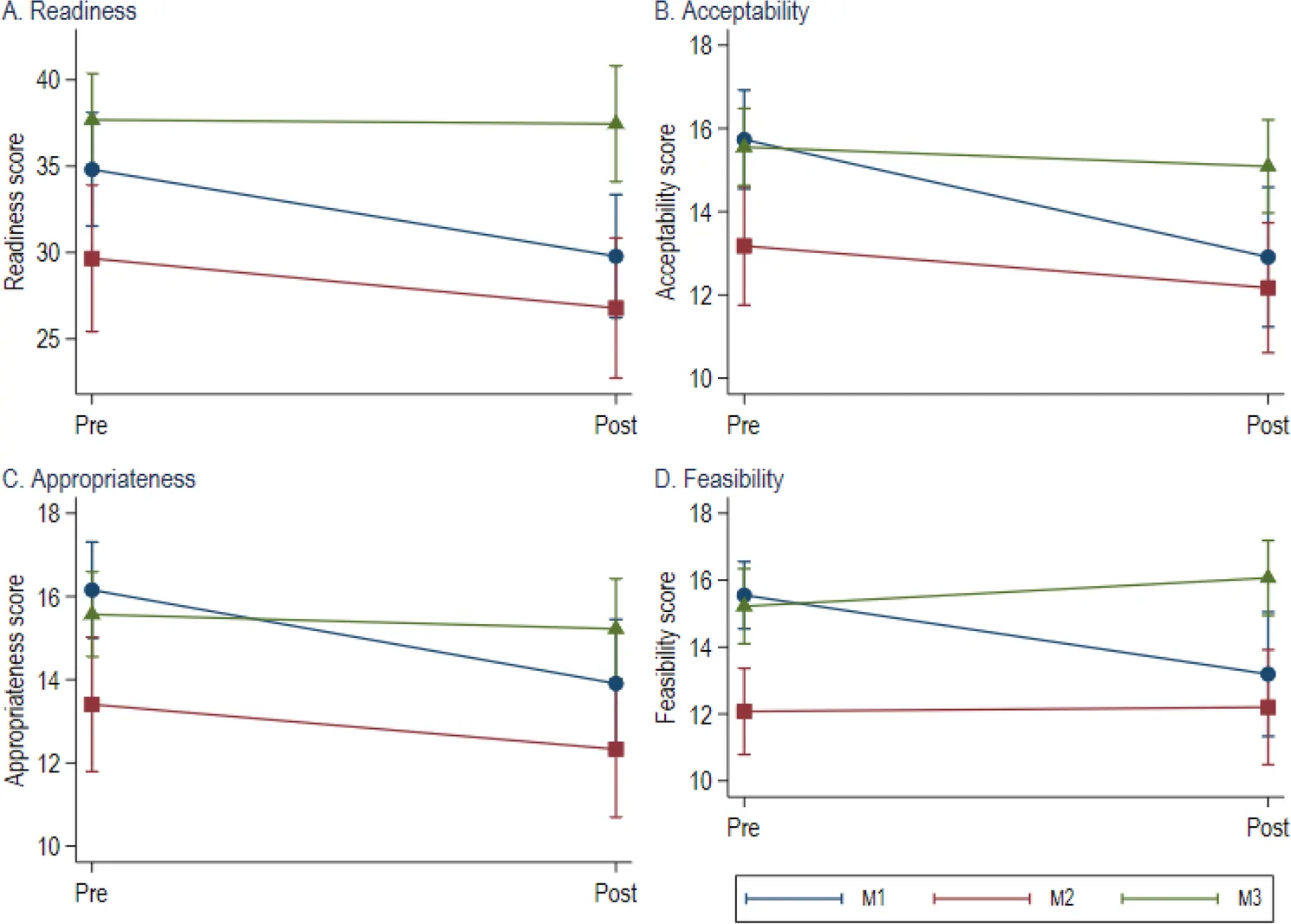
Model-estimated mean scores (with 95% confidence intervals) for readiness (**A**), acceptability (**B**), appropriateness (**C**), and feasibility (**D**) at pre- and post-intervention, by municipality, from multilevel linear regression models. Note that readiness (panel A) is shown on a different y-axis scale than the other three outcomes due to differing instrument ranges. M1–M3 = Municipalities 1–3

Model-estimated mean scores with 95% confidence intervals for all four outcomes at pre- and post-intervention, by municipality, are presented in Fig. 2. Municipality-specific regression results are summarized in Table 3. Descriptive medians by municipality and professional group are provided in Supplementary Tables S1 and S2, and professional-group-specific regression results are reported in Supplementary Table S3. For each outcome, observed median scores are described first, followed by adjusted estimates of pre-to-post change and tests of differences across municipalities and professional groups (Table 3).

**Table 3:** Changes in implementation outcomes from pre- to post-intervention by municipality (M), from multilevel linear regression models

	β	95% CI	P
Readiness (N obs = 248, N ind = 157, N schools = 47)			
Overall pre-to-post change	−2.54	−4.86, -0.22	**0.032**
Change in M1	−5.46	−9.24, −1.67	**0.005**
Change in M2	−2.90	−8.41, 2.61	0.302
Change in M3	−0.22	−3.39, 2.95	0.892
Time × Municipality interaction (χ²(2) = 4.45)			0.108
Time × Professional group interaction (χ²(2) = 0.28)			0.869
Acceptability (N obs = 262, N ind = 161, N schools = 47)			
Overall pre-to-post change	−1.31	−2.21, −0.42	**0.004**
Change in M1	−2.78	−4.41, −1.15	**0.001**
Change in M2	−1.00	−3.12, 1.12	0.356
Change in M3	−0.49	−1.54, 0.56	0.362
Time × Municipality interaction (χ²(2) = 5.52)			0.063
Time × Professional group interaction (χ²(2) = 1.00)			0.606
Appropriateness (N obs = 258, N ind = 160, N schools = 47)			
Overall pre-to-post change	−1.12	−2.08, −0.16	**0.022**
Change in M1	−2.23	−4.09, −0.37	**0.019**
Change in M2	−1.09	−3.22, 1.05	0.318
Change in M3	−0.37	−1.56, 0.82	0.542
Time × Municipality interaction (χ²(2) = 2.84)			0.242
Time × Professional group interaction (χ²(2) = 0.82)			0.665
Feasibility (N obs = 258, N ind = 160, N schools = 47)			
Overall pre-to-post change	−0.30	−1.17, 0.57	0.501
Change in M1	−2.37	−3.97, −0.77	**0.004**
Change in M2	+0.18	−1.46, 1.81	0.831
Change in M3	+0.84	−0.49, 2.18	0.215
Time × Municipality interaction (χ²(2) = 9.56)			**0.008**
Time × Professional group interaction (χ²(2) = 3.64)			0.162

Estimates from multilevel linear regression models with time (pre vs. post), municipality, professional group, and interactions between time and municipality, and time and professional group, as fixed effects. Random intercepts for school and individual. Robust standard errors clustered at school level. Municipality-specific changes computed as marginal effects (margins kom, dydx(time1)). Reference category: Municipality 1. Where the joint time × municipality interaction was not statistically significant, individual municipality-specific estimates should be interpreted descriptively. For feasibility, the significant time × municipality interaction (χ²(2) = 9.56, p = 0.008) indicates that pre-post changes differed formally across municipalities. Professional-group-specific changes are reported in Supplementary Table S3. Bold values indicate statistical significance at p < 0.05

#### Readiness

Median readiness among all personnel declined from 38 (IQR: 26, 43) at baseline to 33 (IQR: 25, 41) post-intervention. By municipality, readiness declined from 38 to 29.5 in M1 and from 28.5 to 27 in M2, while remaining unchanged at 39 in M3. Among professional groups, school nurses reported the lowest median readiness at both time points (baseline: 33.5; post-intervention: 28.5) compared with teachers (baseline: 39; post-intervention: 36) and principals (baseline: 38; post-intervention: 37) (Supplementary Table S2).

In the adjusted model, the overall pre-to-post change in readiness was − 2.54 points (95% CI: −4.86, -0.22; *p* = 0.032). Readiness declined significantly in M1 (adjusted mean change = − 5.46, 95% CI: −9.24, − 1.67; *p* = 0.005), while changes in M2 (− 2.90, 95% CI: −8.41, 2.61; *p* = 0.302) and M3 (− 0.22, 95% CI: −3.39, 2.95; *p* = 0.892) were not statistically significant. Neither interaction was statistically significant (time × municipality: χ²(2) = 4.45, *p* = 0.108; time × professional group: χ²(2) = 0.28, *p* = 0.869). At baseline, M2 scored marginally lower than M1 on readiness (29.7 vs. 34.8, *p* = 0.054).

#### Acceptability (ACC)

Median ACC declined from 15 (IQR: 12, 18) at baseline to 14 (IQR: 10, 17) post-intervention. By municipality, ACC declined sharply in M1 (from 16 to 12) and slightly in M2 (from 12 to 11.5), while remaining stable at 16 in M3. Among professional groups, median ACC scores were similar across roles at both time points (principals: 15 at both; teachers: 16 to 14; school nurses: 15 at both; Supplementary Table S2).

In the adjusted model, the overall pre-to-post change in ACC was − 1.31 points (95% CI: −2.21, − 0.42; *p* = 0.004), a statistically significant decline. The decline was largest in M1 (− 2.78, 95% CI: −4.41, − 1.15; *p* = 0.001), with non-significant changes in M2 (− 1.00, 95% CI: −3.12, 1.12; *p* = 0.356) and M3 (− 0.49, 95% CI: −1.54, 0.56; *p* = 0.362). Neither interaction was statistically significant (time × municipality: χ²(2) = 5.52, *p* = 0.063; time × professional group: χ²(2) = 1.00, *p* = 0.606). At baseline, M2 scored significantly lower than M1 (13.2 vs. 15.7, *p* = 0.009).

#### Appropriateness (APP)

Median APP declined from 16 (IQR: 13, 18) at baseline to 14 (IQR: 11, 17) post-intervention. By municipality, appropriateness declined in M1 (from 16 to 14), shifted minimally in M2 (from 12.5 to 13) and remained stable in M3 (16). Among professional groups, median APP was comparable across roles at baseline (all three groups: 16); post-intervention, teachers (14) and school nurses (14) showed slight declines while principals remained stable (16) (Supplementary Table S2).

In the adjusted model, the overall pre-to-post change in APP was − 1.12 points (95% CI: −2.08, − 0.16; *p* = 0.022), a statistically significant decline. The decline was statistically significant in M1 (− 2.23, 95% CI: −4.09, − 0.37; *p* = 0.019) but not in M2 (− 1.09, 95% CI: −3.22, 1.05; *p* = 0.318) or M3 (− 0.37, 95% CI: −1.56, 0.82; *p* = 0.542). Neither interaction was statistically significant (time × municipality: χ²(2) = 2.84, *p* = 0.242; time × professional group: χ²(2) = 0.82, *p* = 0.665). At baseline, M2 scored significantly lower than M1 (13.5 vs. 16.2, *p* = 0.009).

#### Feasibility (FEAS)

Median FEAS remained stable at 15 from baseline (IQR: 12, 17) to post- intervention (IQR: 10, 18). By municipality, FEAS declined in M1 (from 16 to 15), remained stable in M2 (at 12), and remained unchanged in M3 (at 16). Among professional groups, school nurses reported the lowest median FEAS scores at both time points (baseline: 14; post-intervention: 13.5), compared with teachers (baseline and post-intervention: 16) and principals (baseline and post-intervention: 16) (Supplementary Table S2).

In the adjusted model, the overall pre-to-post change in FEAS was − 0.30 points (95% CI: −1.17, 0.57; *p* = 0.501), not statistically significant. However, the time × municipality interaction was statistically significant (χ²(2) = 9.56, *p* = 0.008), indicating that pre-post changes in FEAS differed across municipalities, while the time × professional group interaction did not (χ²(2) = 3.64, *p* = 0.162). FEAS declined significantly in M1 (− 2.37, 95% CI: −3.97, − 0.77; *p* = 0.004) but remained stable in M2 (+ 0.18, 95% CI: −1.46, 1.81; *p* = 0.831) and M3 (+ 0.84, 95% CI: −0.49, 2.18; *p* = 0.215). At baseline, M2 scored significantly lower than M1 (12.1 vs. 15.5, *p* < 0.001).

### Correlations between baseline readiness and implementation outcomes

**Fig. 3:**
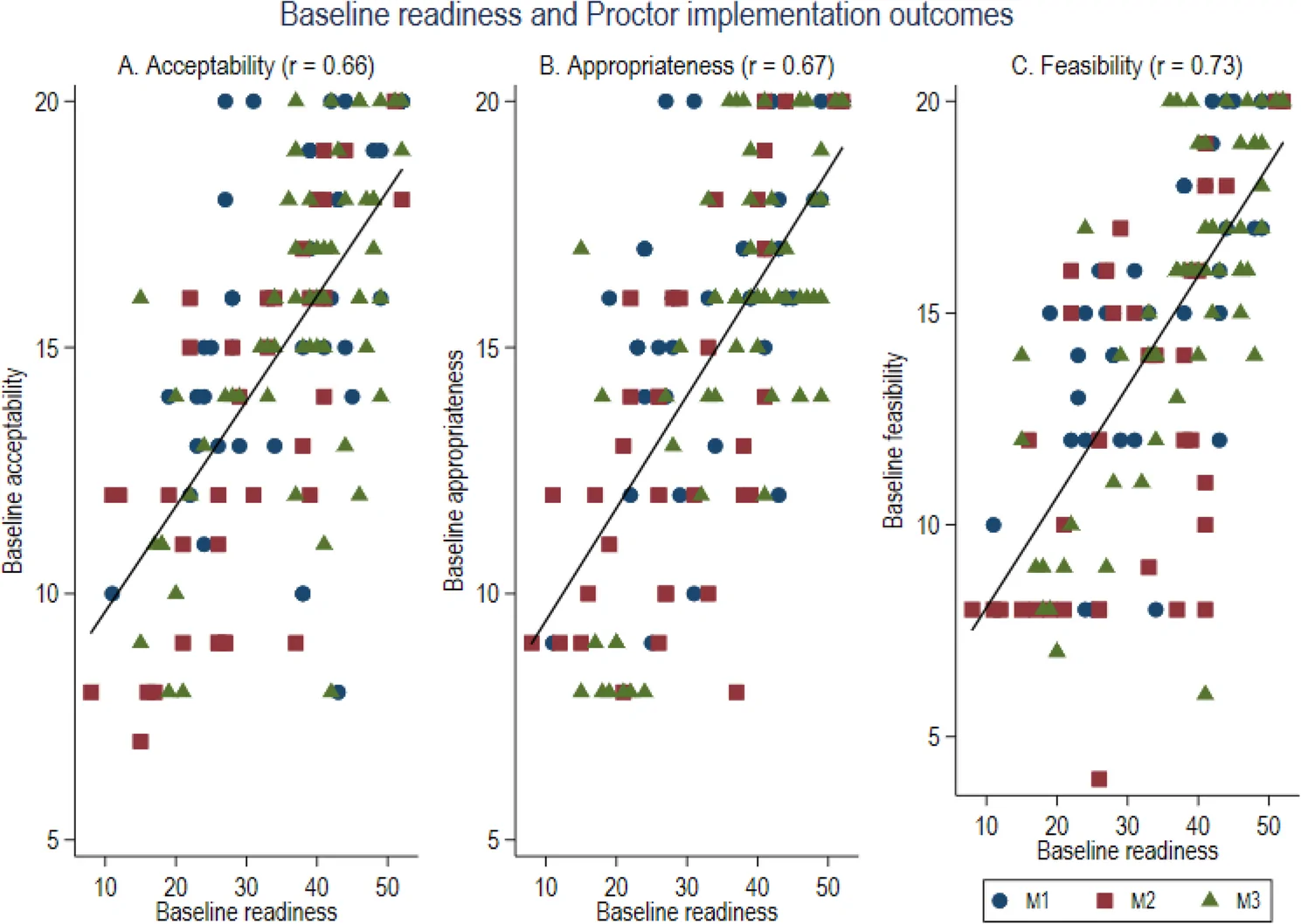
Correlations between baseline Readiness and Acceptability (**A**), Appropriateness (**B**), and Feasibility (**C**) among school personnel in the three municipalities. M1–M3 = Municipalities 1–3

Baseline readiness for all school personnel was positively correlated with baseline ACC (ρ = 0.66, 95% CI: 0.56–0.76, *p* < 0.001), APP (ρ = 0.67, 95% CI: 0.57–0.78, *p* < 0.001), and FEAS (ρ = 0.73, 95% CI: 0.65–0.82, *p* < 0.001) as shown in Fig. 3.

## Discussion

This study examined changes in readiness and the implementation outcomes ACC, APP, and FEAS among school personnel implementing the HSS program across 47 schools in three municipalities. Contrary to expectations, perceived readiness, ACC and APP declined significantly from pre- to post-intervention, while FEAS remained stable overall. Declines were most pronounced in M1, which showed significant reductions across all four outcomes including FEAS. M3, which reported the highest baseline scores, was the only municipality in which scores remained stable across all four outcomes and also the only municipality in which all schools continued the program into the following year. Notably, FEAS was the only outcome for which the pre-post change differed significantly across municipalities, reflecting the divergent trajectories in M1 versus M2 and M3. These patterns suggest that perceptions of readiness and implementation outcomes are not fixed but evolve as personnel gain direct experience of delivery and that context, particularly organizational stability and the conditions under which staff sustain the program, shapes how those perceptions evolve [13].

### Distinct patterns in implementation outcomes between municipalities

One important contextual difference was that implementation began in August 2021, during the COVID-19 pandemic, in M1 and M2, whereas M3 began one year later under more normal conditions. Although no primary schools closed in Sweden during the pandemic, parents were strongly discouraged from entering school buildings [29], so personnel in M1 and M2 implemented the program under constrained and unprecedented circumstances. Despite this shared context, the two municipalities diverged in their outcomes. M1 continued the program in the following year, though not in all schools, and showed significant within-municipality declines across all four outcomes, whereas M2 discontinued it entirely after year one. Evidence of significantly differing trajectories across municipalities was observed only for feasibility. Therefore, the patterns described for readiness, ACC, and APP are descriptive rather than tests of municipality-level differences. Interpreted descriptively, M1’s declines may reflect how the lived experience of sustaining a program under challenging conditions can erode initial perceptions, rather than lower baseline optimism alone. M2’s apparent stability, by contrast, should be interpreted cautiously. It may reflect early disengagement rather than maintained perceptions, and may be further influenced by differential response at follow-up if staff with more negative experiences were less likely to respond. M2’s significantly lower baseline scores also mean there was less scope for further decline than in M1, which may partly account for the apparent stability of M2’s scores over time. In M3, where schools started to implement the program in August 2022, work routines had resumed to more normal conditions. Despite greater socioeconomic disadvantage and poorer health indicators, M3 demonstrated the highest and most stable implementation outcomes.

The fact that it had the highest teacher density per student and higher organizational stability than in M1 and M2 could have contributed to this. This also aligns with previous Swedish research linking organizational stability to implementation success [30]. This is especially true for readiness which appears to be embedded in structural conditions, including funding, workforce stability, policy environments, and population health needs [9]. Committed municipal leadership is important, as it can prioritize and create conditions that favor implementation of health promotion programs like the HSS, as illustrated by M3. In our qualitative study of decision-makers in these municipalities, dedicated municipality leaders who prioritized the program, made it mandatory and provided additional support, emerged as critical for adoption and sustainment [31]. This could help reduce health inequalities within and between municipalities. Continued monitoring of organizational change will be important, as implementation is more likely to succeed in stable organizations able to retain knowledgeable, motivated leaders and staff [32].

### Implementation outcomes between school personnel

Professional groups did not differ significantly in their pre-post scores for any of the four outcomes. However, as a descriptive observation, school nurses consistently reported lower readiness and feasibility scores than teachers and principals at both time points. Although not statistically significant, this pattern is consistent with previous studies of school-based health promotion in which school nurses report a greater implementation burden than other staff [5, 8]. We hypothesize that the additional responsibilities assigned to school nurses in the HSS program, particularly the individual motivational-interviewing health talks with parents, may plausibly contribute to their lower readiness and feasibility scores. This hypothesis is supported by our previous qualitative work, which found that mastering motivational interviewing was initially challenging for school nurses, though with practice they appreciated its ability to promote a more equal power balance with parents [33]. Teachers, by contrast, engage with content closely aligned with the curriculum and process evaluations of the HSS program indicate that teachers value the program strengthening their own health competence [34] and collaboration with parents [7].

### Implementation outcomes and readiness were correlated

Pre-intervention readiness was strongly and positively correlated with ACC, APP, and FEAS, suggesting consistency in how individuals perceive readiness and implementation outcomes at baseline. Another study also found a high correlation between readiness and implementation outcomes and suggested that readiness is a precursor of early implementation outcomes [35]. Those authors warned against implementing a service in an organization with a low appropriateness score without first engaging in a process to improve readiness. Although this overlap raises questions about whether separately measuring all four constructs is always necessary when measurement burden is a concern, the differing patterns observed over time suggest they capture distinct aspects of implementation once exposure begins. Our findings underscore the need to better understand how initial readiness shapes subsequent implementation processes and whether low baseline readiness predicts later declines in implementation perceptions, particularly in unstable organizational contexts such as M1. Future analyses could examine how readiness is associated with more distal implementation outcomes such as fidelity, consistent with the CFIR Outcomes Addendum, which positions readiness, ACC, APP, and FEAS as antecedent assessments on the pathway between implementation determinants and outcomes [36].

### Strengths and limitations

To our knowledge, this is one of the first school-based implementation studies to use the validated questionnaire by Weiner et al. on implementation outcomes [12]. While previous studies have relied primarily on mixed methods to examine acceptability, appropriateness, and feasibility, often in rural school settings [37, 38], the present study applies a standardized, theory-informed quantitative instrument in an urban school-based implementation context. Moreover, a validation study of the readiness instrument in the Swedish context is currently under peer review (A Toropova et al., submitted for publication). Until this validation is published, the reliability and validity evidence for the Swedish version should be considered preliminary. In contrast to the ORIC instrument on which it is based, the readiness scales used in this study ask for a personal appraisal of readiness instead of an assessment of organizational readiness as a whole. Assessing organizational readiness may be challenging for individual staff members, and in this sense, the readiness scale captures individual perceptions of readiness to implement the HSS program, rather than the organization’s readiness per se. The same individual-level perspective applies to the instrument used to assess implementation outcomes. Additional strengths of the study include the repeated-measures design capturing pre- and post-intervention perceptions from the same individuals, the use of multilevel linear regression models with random intercepts for both school and individual which is appropriate for the clustered and repeated-measures structure of the data, and the inclusion of three municipalities with meaningfully different socioeconomic and organizational contexts, allowing implementation patterns to be examined across real-world variation in setting.

A limitation of this study is the relatively small cluster size and sample size within professional subgroups, combined with potential selection bias, as only 66% (151/229) of eligible personnel participated and 74% of these (111/151) completed the post-intervention survey reflecting differential participation at post-intervention. Multilevel linear regression models handle missing data under the assumption of missing at random. While the validity of this assumption cannot be fully verified, we compared completers and non-completers on available baseline characteristics (age, sex, professional role, and municipality) and found no statistically significant differences. The limited statistical power may explain the absence of statistically significant differences in readiness scores between professional groups. Additionally, the mean of 1.6 observations per individual reflects that many participants provided data at only one time point, meaning the individual-level random intercept was estimated from relatively sparse repeated-measures data. This limits precision in estimating within-person variance, and individual-level model parameters should be interpreted with caution. Nonetheless, the inclusion of principals, teachers, and school nurses offers important triangulation across professional roles and provides insight into the additional workload and stress experienced by school nurses when delivering the MI component for the first time. Finally, this study examined a single health promotion program (HSS) in one country (Sweden), delivered within a specific policy and organizational context. Findings may not generalize directly to other programs, other educational systems, or countries with different school health infrastructures, although the general pattern of implementation perceptions evolving with direct experience may have broader relevance. Future studies with larger samples could further explore higher-level organizational and systematic barriers and facilitators to implementation at scale. 

## Conclusion

This study provides novel empirical insight into school personnel’s changing perceptions of preparedness to implement a new health program. Contrary to typical assumptions that exposure improves perceived readiness and implementation outcomes, readiness, acceptability, and appropriateness all declined significantly across the first year of implementation, while feasibility remained stable overall. Declines were most pronounced in M1. Findings suggest that perceptions of implementability vary across local contexts and over time, and that low baseline readiness may indicate a need for additional preparation time or implementation support before full-scale implementation. Baseline readiness was strongly correlated with acceptability, appropriateness, and feasibility, indicating that these constructs are closely aligned at the outset of implementation and reinforcing the value of assessing readiness as an early indicator of implementation potential. By capturing pre- and post-intervention data using validated implementation measures, this study highlights the importance of addressing context-specific barriers before and during implementation rather than assuming exposure alone will strengthen perceptions of implementability.

## Supplementary Information

Supplementary Material 1.

Supplementary Material 2.

Supplementary Material 3.

Supplementary Material 4.

## Data Availability

The datasets used and/or analyzed during the current study are available from the corresponding author on reasonable request.

## Abbreviations

ACC: Acceptability
APP: Appropriateness
FEAS: Feasibility
HSS: Healthy School Start
IMPROVE: IMplementation and evaluation of the school-based family support PRogram a Healthy School Start to promote child health and prevent OVErweight and obesity
IQR: Interquartile Range
LRIT: Leader Readiness to Implement Tool
M: Municipality
ORIC: Organizational Readiness for Implementing Change
REDCap: Research Electronic Data Capture
SRIT: Staff Readiness to Implement Tool
