# Supplementary material for: A multi-site study of implementing a school-based health promotion intervention in Sweden – changes in readiness and implementation outcomes
Source: BMC Public Health. 2026 Sep 8;26:2548. doi: 10.1186/s12889-026-29302-z (PMC13555921; doi:10.1186/s12889-026-29302-z)
Supplement: Supplementary file 1 [file 12889_2026_29302_MOESM1_ESM.docx]

Additional file 1: Pre-implementation activities and timeline in the three municipalities

| Activity | Date | Stakeholder |
| --- | --- | --- |
| 1^st^ online workshop to identify barriers and facilitators to implementation | Fall 2020 | School and primary health care representatives from M1 and M2 |
| 2^nd^ online workshop to discuss and decide implementation strategies based on barriers and facilities from the 1^st^ workshop | Spring 2021 | School representatives from M1 and M2 |
| Introduction of each professional group to the HSS program and IMPROVE study by research team | Spring 2021 | Principals, teachers, and school nurses M1 and M2 |
| Online 2-day training in Motivational Interviewing (MI) adapted for the HSS program | Spring 2021 | School nurses in M1 and M2 |
| Onsite 2-day MI training in Motivational Interviewing adapted for the HSS program | Spring 2022 | School nurses in M3 |
| Introduction of each professional group to the HSS program and IMPROVE study by research team | Spring 2022 | Principals, teachers, and school nurses from M3 |

M: Municipality; HSS: Healthy School Start; IMPROVE: IMplementation and evaluation of the school-based family support PRogram a Healthy School Start to promote child health and prevent OVErweight and obesity
