# Supplementary material for: A multi-site study of implementing a school-based health promotion intervention in Sweden – changes in readiness and implementation outcomes
Source: BMC Public Health. 2026 Sep 8;26:2548. doi: 10.1186/s12889-026-29302-z (PMC13555921; doi:10.1186/s12889-026-29302-z)
Supplement: Supplementary file 2 [file 12889_2026_29302_MOESM2_ESM.docx]

Additional file 2: Acceptability (ACC), Appropriateness (APP) and Feasibility (FEAS) Tool

The following questions regard your opinion about the Healthy School Start Program (Program=En Frisk Skolstart) as you think RIGHT NOW.

**Acceptability**

|  | Completely disagree | Disagree | Neutral | Agree | Completely agree |
| --- | --- | --- | --- | --- | --- |
| 1. HSS Program meets my approval. | 1 | 2 | 3 | 4 | 5 |
| 2. HSS Program is appealing to me. | 1 | 2 | 3 | 4 | 5 |
| 3. I like HSS Program. | 1 | 2 | 3 | 4 | 5 |
| 4. I welcome HSS Program. | 1 | 2 | 3 | 4 | 5 |

**Appropriateness**

|  | Completely disagree | Disagree | Neutral | Agree | Completely agree |
| --- | --- | --- | --- | --- | --- |
| 1. HSS Program seems fitting. | 1 | 2 | 3 | 4 | 5 |
| 2. HSS Program seems suitable. | 1 | 2 | 3 | 4 | 5 |
| 3. HSS Program seems applicable. | 1 | 2 | 3 | 4 | 5 |
| 4. HSS Program seems like a good match. | 1 | 2 | 3 | 4 | 5 |

**Feasibility**

|  | Completely disagree | Disagree | Neutral | Agree | Completely agree |
| --- | --- | --- | --- | --- | --- |
| 1. HSS Program seems implementable. | 1 | 2 | 3 | 4 | 5 |
| 2. HSS Program seems possible. | 1 | 2 | 3 | 4 | 5 |
| 3. HSS Program seems doable. | 1 | 2 | 3 | 4 | 5 |
| 4. HSS Program seems easy to use. | 1 | 2 | 3 | 4 | 5 |
