# Supplementary material for: A multi-site study of implementing a school-based health promotion intervention in Sweden – changes in readiness and implementation outcomes
Source: BMC Public Health. 2026 Sep 8;26:2548. doi: 10.1186/s12889-026-29302-z (PMC13555921; doi:10.1186/s12889-026-29302-z)
Supplement: Supplementary file 3 [file 12889_2026_29302_MOESM3_ESM.docx]

Additional file 3: Readiness Tool – LRIT and SRIT

**Leader Readiness to Implement Tool (LRIT)**

| 0 | 1 | 2 | 3 | 4 |
| --- | --- | --- | --- | --- |
| Disagree | Somewhat Disagree | Neutral | Somewhat Agree | Agree |

You are a formal leader in the district who has the authority and power to make decisions that impact how staff practice. You are preparing for an upcoming change that will involve the implementation of a specific program, practice, and procedure. Reflect on where you are with regard to implementing this change. Be honest as possible as these data are anonymous and will be aggregated up to understand where the organization as whole is at with being ready to implement this change.

| 1. I feel confident in my ability to get staff invested in implementing this change. | 0 1 2 3 4 |
| --- | --- |
| 2. I feel confident in my ability to keep track of progress in implementing this change. | 0 1 2 3 4 |
| 3. I will do whatever it takes to implement this change. | 0 1 2 3 4 |
| 4. I feel confident in my ability to support staff as they adjust to this change. | 0 1 2 3 4 |
| 5. I want to implement this change. | 0 1 2 3 4 |
| 6. I feel confident that I can maintain momentum over time with implementing this change. | 0 1 2 3 4 |
| 7. I feel confident in my ability to handle the challenges that might arise in implementing this change. | 0 1 2 3 4 |
| 8. I am determined to implement this change. | 0 1 2 3 4 |
| 9. I feel capable to coordinate tasks so that implementation goes smoothly. | 0 1 2 3 4 |
| 10. I am motivated to commit time and energy to implement this change. | 0 1 2 3 4 |
| 11. I feel confident I can manage the politics of implementing this change. | 0 1 2 3 4 |
| 12. I will devote time to collaborate with other leaders to action plan implementing this change. | 0 1 2 3 4 |
| 13. I intend to prioritize the implementation of this change. | 0 1 2 3 4 |

**Staff Readiness to Implement (SRIT)**

| 0 | 1 | 2 | 3 | 4 |
| --- | --- | --- | --- | --- |
| Disagree | Somewhat Disagree | Neutral | Somewhat Agree | Agree |

You are a staff member in the district who ultimately spends time interfacing with students and. You are preparing for an upcoming change that will involve the implementation of a specific program, practice, and procedure. Reflect on where you are with regard to implementing this change. Be honest as possible as these data are anonymous and will be aggregated up to understand where the organization as whole is at with being ready to implement this change.

| 1. I feel confident in my ability to implement this change. | 0 1 2 3 4 |
| --- | --- |
| 2. I feel confident in my ability to keep track of my progress in implementing this change. | 0 1 2 3 4 |
| 3. I will do whatever it takes to implement this change. | 0 1 2 3 4 |
| 4. I feel confident in my ability to adjust how I practice to implement this change. | 0 1 2 3 4 |
| 5. I want to implement this change. | 0 1 2 3 4 |
| 6. I feel confident that I can maintain momentum over time with implementing this change. | 0 1 2 3 4 |
| 7. I feel confident in my ability to handle the challenges that might arise in implementing this change. | 0 1 2 3 4 |
| 8. I am determined to implement this change. | 0 1 2 3 4 |
| 9. I feel capable to coordinate tasks so that implementation goes smoothly. | 0 1 2 3 4 |
| 10. I am motivated to commit time and energy to implement this change. | 0 1 2 3 4 |
| 11. I feel confident I can manage implementing this change with other things that are on my plate. | 0 1 2 3 4 |
| 12. I will devote time to collaborate with others to action plan implementing this change. | 0 1 2 3 4 |
| 13. I intend to prioritize the implementation of this change. | 0 1 2 3 4 |
