# Supplementary material for: A multi-site study of implementing a school-based health promotion intervention in Sweden – changes in readiness and implementation outcomes
Source: BMC Public Health. 2026 Sep 8;26:2548. doi: 10.1186/s12889-026-29302-z (PMC13555921; doi:10.1186/s12889-026-29302-z)
Supplement: Supplementary file 4 [file 12889_2026_29302_MOESM4_ESM.docx]

**Supplementary Table S1. Median (interquartile range) scores for implementation outcomes by municipality at pre- and post-intervention**

| **Outcome** | **Time** | **Municipality 1** | **Municipality 2** | **Municipality 3** |
| --- | --- | --- | --- | --- |
| **Readiness** | Pre | 38 (27, 43) | 28.5 (22, 39) | 39 (33, 46) |
|  | Post | 29.5 (21.5, 43.5) | 27 (23.5, 34) | 39 (33, 44) |
| **Acceptability** | Pre | 16 (14, 19) | 12 (10, 16) | 16 (14, 18) |
|  | Post | 12 (9, 16) | 11.5 (10, 16) | 16 (13, 18) |
| **Appropriateness** | Pre | 16 (15, 18) | 12.5 (10, 16.5) | 16 (14, 18) |
|  | Post | 14 (11, 18) | 13 (9, 15) | 16 (14, 17.5) |
| **Feasibility** | Pre | 16 (14, 18) | 12 (8, 16) | 16 (14, 17) |
|  | Post | 15 (9, 17) | 12 (8.5, 16) | 16 (14.5, 19) |

*Notes: Values are medians (interquartile range). Sample sizes vary slightly across outcomes due to item-level missingness (see Table 3 for outcome-specific Ns). Readiness score range: 0–52; Acceptability, Appropriateness, Feasibility score range: 4–20*

**Supplementary Table S2. Median (interquartile range) scores for implementation outcomes by professional group at pre- and post-intervention**

| **Outcome** | **Time** | **Principals** | **Teachers** | **School nurses** |
| --- | --- | --- | --- | --- |
| **Readiness** | Pre | 38 (29, 42) | 39 (26, 45) | 33.5 (23, 41) |
|  | Post | 37 (25, 40) | 36 (25, 44) | 28.5 (24, 40) |
| **Acceptability** | Pre | 15 (12, 18) | 16 (12.5, 18) | 15 (10.5, 16) |
|  | Post | 15 (11, 17.5) | 14 (10, 17) | 15 (11, 16) |
| **Appropriateness** | Pre | 16 (14, 17) | 16 (12, 18) | 16 (12, 17) |
|  | Post | 16 (12, 19) | 14 (11, 17) | 14 (9.5, 16) |
| **Feasibility** | Pre | 16 (14, 18) | 16 (12, 18) | 14 (10, 16) |
|  | Post | 16 (11, 20) | 16 (12, 19) | 13.5 (8.5, 16) |

*Notes: Values are medians (interquartile range). Sample sizes vary slightly across outcomes due to item-level missingness (see Table 3 for outcome-specific Ns). Readiness score range: 0–52; Acceptability, Appropriateness, Feasibility score range: 4–20*

**Supplementary Table S3. Professional-group-specific changes from pre- to post-intervention, from multilevel linear regression models**

|  | ***β*** | ***95% CI*** | ***p*** |
| --- | --- | --- | --- |
| **Readiness** (N obs = 248, N ind = 157, N schools = 47) |  |  |  |
| Change in Principals | −3.62 | −8.10, 0.85 | 0.112 |
| Change in Teachers | −2.08 | −5.81, 1.66 | 0.276 |
| Change in School nurses | −2.32 | −5.82, 1.17 | 0.193 |
| Time × Professional group interaction (χ²(2) = 0.28) |  |  | 0.869 |
| **Acceptability**  (N obs = 262, N ind = 161, N schools = 47) |  |  |  |
| Change in Principals | −1.62 | −2.92, −0.32 | **0.014** |
| Change in Teachers | −1.48 | −2.86, −0.09 | **0.036** |
| Change in School nurses | −0.75 | −2.11, 0.60 | 0.277 |
| Time × Professional group interaction (χ²(2) = 1.00) |  |  | 0.606 |
| **Appropriateness** (N obs = 258, N ind = 160, N schools = 47) |  |  |  |
| Change in Principals | −0.90 | −2.37, 0.57 | 0.229 |
| Change in Teachers | −0.93 | −2.37, 0.51 | 0.208 |
| Change in School nurses | −1.66 | −3.09, −0.23 | **0.023** |
| Time × Professional group interaction (χ²(2) = 0.82) |  |  | 0.665 |
| **Feasibility** (N obs = 258, N ind = 160, N schools = 47) |  |  |  |
| Change in Principals | −1.14 | −2.71, 0.43 | 0.155 |
| Change in Teachers | +0.56 | −0.83, 1.95 | 0.432 |
| Change in School nurses | −0.98 | −2.54, 0.57 | 0.215 |
| Time × Professional group interaction (χ²(2) = 3.64) |  |  | 0.162 |

*Note: Within-group pre-to-post changes from the multilevel models reported in Table 3, computed as marginal effects (margins cat_base, dydx(time1)). Reference category: Principals. The joint time × professional group interaction was not statistically significant for any outcome, indicating no formal evidence of differences in pre-post trajectories between professional groups. Within-group estimates should therefore be interpreted descriptively. Bold values indicate p < 0.05*
